# The Evolution of Targeted Radionuclide Diagnosis of HER2-Positive Breast Cancer

**DOI:** 10.32607/actanaturae.11611

**Published:** 2022

**Authors:** O. D. Bragina, S. M. Deyev, V. I. Chernov, V. M. Tolmachev

**Affiliations:** Tomsk National Research Medical Center of the Russian Academy of Sciences Cancer Research institute, Tomsk, 634009 Russia; National Research Tomsk Polytechnic University, Tomsk, 634050 Russia; Shemyakin-Ovchinnikov Institute of Bioorganic Chemistry of the Russian Academy of Sciences, Moscow, 117997 Russia; Uppsala University, Uppsala, Sweden

**Keywords:** breast cancer, HER2/neu, radionuclide diagnostics, monoclonal antibody, alternative scaffold proteins

## Abstract

This review examines the evolution of the radionuclide diagnosis of HER2-positive breast cancer using various compounds as a targeting module in clinical practice: from full-length antibodies to a new group of small synthetic proteins called alternative scaffold proteins. This topic is of especial relevance today in view of the problems attendant to the detection of breast cancer with HER2/neu overexpression, which, in most cases, introduce errors in the treatment of patients. The results of clinical studies of radiopharmaceuticals based on affibody molecules, ADAPTs, and DARPins for SPECT and PET have demonstrated good tolerability of the compounds, their rapid excretion from the body, and the possibility to differentiate tumor sites depending on the HER2/neu status. This indicates that targeted radionuclide diagnosis holds promise and the need to continue research in this direction.

## INTRODUCTION

More than 10 million new cancer cases are diagnosed annually in the world, with about 7.6 million people dying from the pathology [1]. Breast cancer (BC) holds a stable, leading position among oncological diseases for women in terms of morbidity and mortality [2]. For instance, according to the ESMO (European Society for Medical Oncology) guidelines, about 2.1 million new BC cases were diagnosed worldwide (almost every fourth case) in 2018, while 630,000 patients died from the pathology [3]. A total of 70,682 BC cases (20.9% of all oncological diseases in women) with a mortality rate of 1.6% were recorded in the Russian Federation in 2019 [4]. BC also takes the first place (16.2%) among death causes in women [5].

Despite the widespread prevalence of BC, the life expectancy of patients diagnosed with it over the past five years or more (patients with or without disease signs) has been increasing since 2012. For instance, this parameter is 59.8% in the Russian Federation [6]. These results primarily have to do with the improvement achieved in diagnostic algorithms, as well as in both local and systemic treatment [7]. In particular, the concept of personalized medicine, which implies the administration of treatment based on the individual characteristics of a patient, with taking into account his/her expected response to it, has gained wide use in the treatment of oncological diseases in recent years [8]. The concept of personalized medicine has become conspicuous in oncological practice. One of the most rapidly developing areas of personalized medicine is theranostics; it combines such concepts as therapy and diagnosis and involves the use of agents and methods based on diagnostic imaging and targeted therapy [9]. The imaging stage of the theranostic approach consists in image processing, visualization of a biological target, and identification of a subgroup of patients in whom the planned treatment is expected to be at its most efficacious; the subsequent therapeutic stage includes the administration of a drug that acts on previously identified targets [10]. The main goals of this strategy are to increase therapy effectiveness, improve the survival rates of cancer patients, reduce adverse reactions and, as a result, decrease overall costs [11]. The rapid progress achieved in the development of theranostics is largely due to the accumulation of new data on the molecular basis of carcinogenesis, the creation of technologies for the manufacture of new biological agents, and the improvement in the performance and accuracy of diagnostic devices [12].

## EPIDERMAL GROWTH FACTOR RECEPTOR HER2/neu

One of the most studied molecular targets located on the surface of cancer cells is the human epidermal growth factor receptor 2 (HER2/neu). It belongs to the family of transmembrane receptors to EGF (epidermal growth factor receptor: ErbB1/HER1, ErbB2/ HER2, ErbB3/HER3, and ErbB4/HER4) tyrosine kinases and regulates the processes of cell division, growth, differentiation, proliferation, migration, and apoptosis [13, 14]. HER2/neu overexpression, which is found in gastric, ovarian, prostate, lung, bladder and other cancers, is most common in invasive BC [15, 16]. In most cases, increased HER2/neu expression in a cancer cell is due to an amplification of the *ERBB2 *gene located in the 17q12 locus of the chromosome and associated with specific changes in some loci of other chromosomes (11q13, 16q22–q24, and 18q21) [17].

Hyperexpression of HER2/neu and/or amplification of *ERBB2 *is observed in 15–20% of BC cases; they are considered an unfavorable prognostic factor and are characterized by an aggressive disease course, as well as low rates of overall and disease-free survival [18, 19]. According to Russian and international clinical guidelines, tumors characterized by a high expression of this receptor require targeted treatment involving drugs used both as monotherapy and in combination with chemotherapy [20]. The targeted drug Herceptin, containing the humanized monoclonal antibody (mAb) trastuzumab, which the first compound capable of suppressing HER2/neu function approved by the FDA (Food and Drug Administration, USA) in 1998, remains the gold standard in the treatment of HER2- positive BC. The use of trastuzumab in combination with taxanes to treat metastatic BC has resulted in an increased response rate, progression-free survival (PFS), and overall survival (OS) [21]. Targeted therapy requires a careful selection of candidates [22]. To date, several methods have been developed to determine the HER2/neu status; they evaluate the protein, DNA, and RNA levels of marker expression. The FDA-approved immunohistochemistry (IHC) and fluorescence* in situ *hybridization (FISH) based methods are the most widespread among them [23].

*The immunohistochemical study (IHC) *is a widely used method used to evaluate HER2/neu expression on a cancer cell surface in formalin-fixed BC samples [24]. According to the 2018 American Society of Clinical Oncology (ASCO) guidelines, 0 and 1+ cases are considered negative, while cases scored as 3+ are considered positive. Targeted therapy is recomended for patients in whom the receptor overexpression corresponds to a score of 3+. Cases 2+ are considered equivocal and require a FISH analysis to verify* ERBB2 *amplification [25].

Despite the availability and relatively low cost of the analysis, IHC results can be significantly affected by numerous factors, such as sample preparation method (duration of fixation and the fixative used), characteristics of the antibodies used (manufacturer), personnel qualifications, and interpretation of the results (mainly cases with a 2+ score) [26].

*Fluorescent in situ hybridization (FISH) *is a cytogenetic technique based on the use of fluorescently labeled probes to detect specific DNA sequences in formalin-fixed BC tissue samples. FISH is used to quantify the *ERBB2 *copy number in the nuclei of BC cells; amplification is considered positive in the presence of an average number of *ERBB2 *copies and an average number of chromosome 17 centromeres per cell of > 2.2. The undeniable advantages of FISH are more objective and quantitative results compared to IHC, which is probably due to greater DNA stability and the presence of internal controls consisting of non-amplified signals in the non-tumor cells (ductal epithelial and stromal cells) adjacent to the tumor [27].

FISH is a very reliable method for evaluating* ERBB2 *amplification. However, it takes nine times longer (36 h vs 4 h); it is several times more expensive than standard ICH and requires expensive equipment to detect and recognize signals, as well as highly qualified personnel to analyze the obtained data [28].

From a clinical standpoint, a significant drawback of conventional methods for determining the HER2/ neu status at the diagnostic stage is the impossibility to simultaneously assess tumor progression *in vivo* and analyze the molecular characteristics of identified tumor lesions prior to the administration of a specific treatment [29]. This fact is of particular importance in light of the increasingly discussed heterogeneity of HER2/neu expression in the primary tumor, local and distant metastatic lesions which can occur, according to various analysis data, in 6–48% of cases [30]. A discrepancy in the HER2/neu status between the primary tumor and affected lymph nodes was revealed in almost 20% of BC cases in [31]. In the case of metastatic lesions in distant organs and tissues, this discrepancy stood at 14.3%, according to Lower et al. but reached 34% in the study by Turner et al. [32, 33]. This fact is most significant in metastatic BC, which is characterized by a long and scallopy course requiring several stages and types of systemic treatment. At the same time, performing a biopsy and surgical sampling from existing and/or newly identified metastatic lesions to optimize the treatment strategy is sometimes either technically impossible or may lead to serious complications [30].

The problem of intratumoral heterogeneity, which is observed in 40% of BC cases and can be represented in the coexistence of many subpopulations of cells with different HER2/neu expression levels in the same tumor, remains unsolved [34, 35]. Recent studies have shown that the RFS and effectiveness of a targeted therapy with trastuzumab are reduced in HER2-positive BC patients with intratumoral heterogeneity of the receptor expression compared to tumors with homogeneous expression [36]. Despite this, the relationship between HER2 heterogeneity and long-term treatment outcomes in patients after surgery remains to be studied. All of this calls for the development of new additional diagnostic techniques in order to optimize the diagnosis of BC [37].

## METHODS FOR RADIONUCLIDE DIAGNOSIS OF HER2-POSITIVE BREAST CANCER

In recent years, the possibility of diagnosing cancer using targeted radionuclide methods has become possible [38]. One of the most studied approaches based on binding to the HER2/neu receptor is the use of labeled monoclonal antibodies (mAbs) [39]. The diagnostic radiopharmaceuticals (RPs) used in oncological practice belong to the category of substances that contain radionuclides for single-photon-emission-computed tomography (SPECT) (γ-emitters with energies in the range of 100–200 keV and half-lives ranging from several minutes to several days) and positron emission tomography (PET) (β+-emitters with halflives ranging from several seconds to several hours) [40]. A comparative characterization of the radioisotopes used for radionuclide imaging is presented in *Table 1*.

**Table 1:** Radioisotopes for radionuclide diagnosis by PET and SPECT

Radioisotope	Half-life, T_1/2_	Production method
Radioisotopes for SPECT
^99m^Tc	6.01 h	Generator
^123^I	13.3 h	Cyclotron
^111^In	2.8 days	Cyclotron
Radioisotopes for PET
^15^O	2.03 min	Cyclotron
^13^N	9.97 min	Cyclotron
^11^C	20.4 min	Cyclotron
^68^Ga	67.7 min	Generator
^18^F	109.8 min	Cyclotron
^64^Cu	12.7 h	Cyclotron
^76^Br	16.2 h	Cyclotron
^89Zr^	78.4 h	Cyclotron
^124^I	100 h	Cyclotron

SPECT has become widespread largely due to its low cost, while PET diagnostics, which is costlier, affords a significantly higher sensitivity, spatial resolution, and quantification accuracy. The recent introduction of scanners for SPECT diagnostics based on cadmium and zinc tellurides can significantly increase camera sensitivity and resolution [41, 42].

Radionuclide imaging of oncological diseases with HER2/neu overexpression has a number of significant advantages compared to invasive diagnostic methods. These advantages include the non-invasive nature of the study with a possibility to conduct repeated studies [43], the assessment of marker expression over time during treatment, simultaneous visualization of a patient’s whole body with an evaluation of HER/neu receptor expression in the primary tumor and metastatic foci, as well as improvement of the diagnostic equipment in the form of developing devices that combine both modules for radionuclide studies and anatomical visualization of metastatic lesions (computed tomography (CT) and magnetic resonance imaging (MRI)) [44].

To date, several types of targeting modules that can be potentially used for radionuclide imaging of HER2/neu receptors are known: monoclonal antibodies; antibody fragments (Fab- and (Fab)2-fragments), diabodies, minibodies, single-chain variable fragments of scFv and nanobodies, nucleic acid aptamers, rationally designed short peptides, and alternative scaffold proteins (ASPs, scaffolds) selected using the molecular display approach (*Table 2*) [45, 46].

**Table 2:** Radionuclide diagnosis of HER2-positive breast cancer (clinical studies)

Protein type	Agent name	Visualization technique	Patient population	Ref.
Full-length antibodies	^111^In-trastuzumab	SPECT/CT	Metastatic breast cancer	[51, 52, 53]
	^89^Zr-trastuzumab	PET/CT	Metastatic breast cancer	[54, 55, 56]
	^64^Cu-trastuzumab	PET/CT	Primary metastatic breast cancer	[57, 58]
Antibody fragments	^68^Ga-DOTA-F(ab′)2-trastuzumab	PET/CT	Metastatic breast cancer	[59]
	^68^Ga-HER2-Nanobody	PET/CT	Metastatic breast cancer	[60]
Alternative scaffold proteins	^111^In-ABY-002	SPECT/CT	Metastatic breast cancer	[61]
	^68^Ga-ABY-002	PET/CT		
	^111^In-ABY-025	SPECT/CT	Locally advanced metastatic breast cancer	[62, 63]
	^68^Ga-ABY-025	PET/CT	Locally advanced metastatic breast cancer	[63, 64, 65]
	^99m^Tc-ADAPT6	SPECT	Operable locally advanced metastatic breast cancer	[66]
	^99m^Tc-DARPinG3	SPECT	Operable locally advanced metastatic breast cancer	[67]

**Radionuclide diagnosis of HER2-positive breast cancer using full-length antibodies**

Full-length monoclonal antibodies labeled with various radioisotopes were the first targeting modules used to evaluate HER2 expression [47]. The highly specific interaction between mAb and the corresponding antigen has become the starting point for preclinical and clinical studies aimed at exploring the possi bility of using antibodies as a capture agent for either delivering radionuclides to tumor cells, visualizing them, or exerting a radiation cytotoxic effect on them. Long-term circulation of mAbs in a patient’s body required the use of long-lived positron emitters such as 89Zr (zirconium-89), ^64^Cu (copper-64), ^124^I (iodine-124), and ^86^Y (yttrium-86) [48].

Since the creation of trastuzumab as a drug to treat BC patients with HER2/neu overexpression, drug molecules labeled with various radioisotopes have been extensively used to study the diagnostic efficiency of HER2 expression evaluation [49]. The drug 111In-trastuzumab (111In; a half-life of 2.8 days) was the first labeled monoclonal antibody clinically tested in HER2-positive BC patients [50]. At first, the cardiotoxicity of the compound for the most part. For instance, Behr et al. studied 20 patients with HER2-positive metastatic BC treated with trastuzumab in 2000. The authors evaluated a potential tumor response to therapy and the possibility to predict cardiotoxicity during treatment. Based on the study results, the authors concluded that the drug could be used as a tool to predict therapeutic efficacy and cardiotoxicity risk during targeted therapy (*Table 2*) [51].

Perik et al. used ^111^In-trastuzumab in 17 patients with metastatic HER2-positive BC. Only one patient with severe cardiotoxicity showed weak uptake of the labeled protein; tumors overexpressing HER2 were detected in 45% of cases, which was an indication of an absence of diagnostic significance for ^111^Intrastuzumab in predicting cardiotoxicity in these patients [52].

Sietske et al. studied 111In-trastuzumab accumulation at the beginning of and 14 weeks after Herceptin therapy in 17 patients with HER2-positive BC. The study results revealed a stable uptake of the drug by all tumor lesions throughout the treatment course, with only a 20% decrease in uptake by the end of the therapy. This analysis showed that the number of HER2 receptor molecules on the cancer cell surface is sufficient for binding to targeted drugs; the decrease in the accumulation was largely due to a reduction in the tumor volume resulting from the combined chemotherapy, as well as competition between circulating “therapeutic” trastuzumab and labeled antibodies for binding to the target receptor. Apparently, the obtained result can be explained by an insufficient dosage of the mAb used and, therefore, incomplete blocking of HER2/neu receptors [53].

The first clinical study of 89Zr-trastuzumab (89Zr; half-life of 78.4 h) conducted in 14 patients with metastatic BC showed a high accumulation of the labeled antibody in the primary tumor and metastatic nodes with a positive HER2/neu status 4–5 days after their injection, according to PET data (anatomical localization of which was comparable to that established by CT and MRI). BC metastases to the brain due to dam age to the blood–brain barrier at the site of the metastasis have also been visualized [54].

The drug 89Zr-trastuzumab was also studied by Ulaner et al. The authors conducted a prospective clinical analysis of 11 patients with HER2-negative BC who had at least one metastatic lesion at the time of the study. Metastatic lesions overexpressing HER2/neu were detected in four out of 11 patients (36%) 5–6 days after drug administration by PET/CT. However, subsequent ICH and FISH analysis of tumor tissue showed that the results were false-positive in three out of the four (75%) identified nodes. It is possible that such a high frequency of false-positive results could be due to nonspecific accumulation of the drug in tumor lesions, because of the large size of its molecules [55].

Gebhart et al. evaluated the possibility of using ^89^Zr-trastuzumab- and 18F-fluorodeoxyglucose- (^18^F-FDG-) PET to assess the efficacy of trastuzumab emtansine (T-DM1) therapy in 56 patients with advanced HER2-positive BC in a multicentric clinical trial (the ZEPHIR study). A total of 16 (29%) patients (with high level of HER2/neu expression in tumor metastases that had been previously diagnosed by IHC) showed no signs of ^89^Zr-trastuzumab accumulation, while co-administration of ^89^Zr-trastuzumab and ^18^F-FDG made it possible to predict the tumor response to treatment in all cases [56].

Tamura et al. and Mortimer et al studied the characteristics and efficacy of ^64^Cu-trastuzumab (^64^Cu; a half-life of 12.7 h). In the first case, a PET study of six patients with either operable or metastatic HER2- positive BC showed the safety of the good visualization of the primary tumor and brain metastases in two patients [57]. Drug effectiveness was confirmed in eight patients with metastatic HER2-positive BC in the study by Mortimer et al. Both the primary tumor and metastatic lesions in the bones, lymph nodes, liver, lungs, and pleura were well visible in all patients [58]. The main disadvantage of ^64^Cu compounds is that their half-life is too short.

Despite the positive results that have been noted in numerous studies, the use of full-length antibodies as a targeting module also revealed some obvious drawbacks. These drawbacks are mainly related to the size of immunoglobulin molecules: slow excretion of mAb from the body, which significantly reduces imaging sensitivity and delays the start of research for 4–7 days after injection; a noticeably higher radiation exposure to patients through the use of longlived radiation sources; slow extravasation and diffusion of drugs to the tumor interstitium, as well as nonspecific accumulation of labeled compounds in the tumor (intake of nonspecific antibodies by the tumor), which results in a high level of false-positive results [68].

**Radionuclide diagnosis of HER2-positive breast cancer using antibody fragments**

The obvious need to modify full-length antibodies (150 kDa) and improve their pharmacokinetics served as a starting point for the synthesis of Fab (~55 kDa) and (Fab)_2_ (~110 kDa) antibody fragments obtained by enzymatic treatment of antibodies with pepsin and papain. These fragments lack an effector function (due to the absence of the Fc domain) and cannot recycle from lysosomes. Like the precursor immunoglobulin, the Fab and (Fab)_2_ fragments are specific to a molecular target and preserve its spatial structure. Both fragments were used for radionuclide imaging of the tumors, which made it possible to evaluate their advantages over full-length antibodies: faster elimination from the bloodstream, compressed time between injection and imaging, decreased absorbed dose for patients, and better contrasting on the day of injection and the next day after injection. This allows for using relatively short-lived radionuclides such as ^99m^Tc (T1/2 = 6.0 h) and positron emitters with an average half-life: ^55^Co (T_1/2_ = 17.5), ^64^Cu (T1/2 = 12.7 h), ^76^Br (T1/2 = 16.2 h), and ^86^Y (T_1/2_ = 14.7 h) [69].

The only drug in this category that has passed phase I clinical trials is ^68^Ga-DOTA-F(ab′)2- trastuzumab, which was administered to 16 patients with metastatic and primary BC with different levels of HER2/neu expression. According to Beylergil et al., the compound was well tolerated by all patients, without pronounced adverse and allergic reactions and demonstrated low sensitivity (50%): the tumor was visualized only in four out of eight HER2-positive patients and not visualized in patients with HER2- negative tumors [59]. Preclinical and clinical studies revealed such shortcomings in this group of drugs as a decrease in the apparent binding affinity compared to monoclonal antibodies and significant sizes for effective extravasation, all things that significantly limit their use in clinical practice.

The discovery of camel heavy-chain-only antibodies (HCAbs) initiated the development of third-generation antibodies consisting of a single heavy chain variable domain (VHH; ~15 kDa) as the antigen-binding region; they were named nanobodies. One of the areas of nanobody application in clinical practice is the molecular imaging of tumors; in particular, their application in nuclear medicine [70, 71]. For instance, the possibility of using ^68^Ga-HER2-Nanobody (halflife; 67.7 min) to detect HER2 receptor expression by PET/CT was evaluated in 20 patients with primary and metastatic BC in a phase I clinical study. Drug safety and the absence of adverse effects at a radiation dose comparable to that of other commonly used PET tracers, as well as its rapid elimination from the bloodstream and accumulation mainly in the kidneys, liver, and intestines, with low accumulation in the area of mammary glands and regional lymph nodes, were shown [60]. Phase II clinical trials of 68Ga-HER2- Nanobody aimed at determining HER2 expression in the brain metastases of BC patients are currently under way [72].

**Radionuclide diagnosis of HER2-positive breast cancer using alternative scaffold proteins**

The search for new effective agents capable of interacting with specific targets, as well as the rapid development of genetic engineering tools, has initiated an intensive effort to study and develop molecular compound alternatives to antibody-binding domains. These compounds must possess a number of necessary characteristics, such as being able to bind exclusively to the target antigen for a specific localization, lack immunogenicity, be stable and able to undergo rapid chemical modification during labeling, rapidly remove unbound molecules from the patient’s body in order to make possible high-quality images of tumor lesions, and permit a reduction of the time interval between the injection and the start of research [73].

Over the past decade, a new class of target molecules called ASPs or scaffolds has gained increasing popularity. They meet all the requirements for optimal radionuclide delivery to tumor cells. The term “scaffold” was first introduced by Plyuktun et al. to designate a protein backbone that makes it possible to obtain various protein variants with different functions by slightly modifying their amino acid sequences and finding variants among them that effectively bind to specific targets [74]. The undeniable advantages of these compounds include their significantly smaller size compared to a conventional antibody, which increases substance penetration into the tumor, as well as their stable structure, additional functionalization and expression in the bacterial system that result in low production costs, and high thermal stability, which allows for long-term storage at room temperature, and the possibility to perform a direct chemical synthesis [75].

**Fig. 1:**
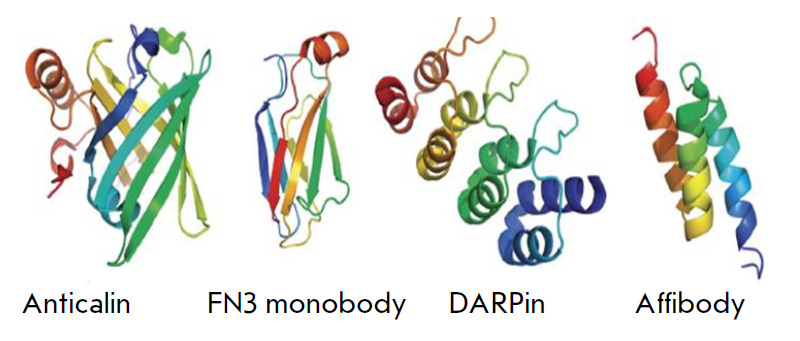
Schematic representation of several alternative scaffold proteins

ASPs can be classified based on various criteria, such as size, synthesis method, origin, and biological function. One of the major classification systems divides scaffold proteins based on their structural elements, which has to do with the possibility of imparting their biological properties to new derivatives. The first class includes domain-sized compounds (6–20 kDa) such as affibody (Affibody, Inc.), albumin-binding-domain-derived affinity proteins (ADAPTs), affilins (Scil Proteins GmbH), anticalins (Pieris Pharmaceuticals Inc.), atrimers (Anaphore Inc.), DARPins (Dyax Inc., Shire Inc.), FN3 scaffolds (Molecular Partners Inc.), fynomer platforms (Janssen), Kunitz-type inhibitor domains and pronectins (Protelica Inc.), and FN3-based sequences (Protelica Inc.). The second class includes constrained peptides (2–4 kDa) such as avimers (Avidia Inc.), bicyclic peptides (Bicycle Therapeutics Inc.), and cysteine- containing peptides. To date, three scaffolds have been clinically tested for the diagnosis of HER2- positive BC: affibodies, ADAPTs, and DARPins (*Fig. 1*) [76].

*Affibodies. *Affibody molecules are composed of three densely packed alpha helices stabilized by a hydrophobic core [77]. Affibodiies are small proteins with a molecular weight of 6–7 kDa that consist of 58 amino-acid residues. Affibodies display high affinity for HER3, IGF-1R, CAIX, and VEGFR2 receptors. Preclinical studies have revealed the high potential of affibodies as targeting modules for radionuclide diagnosis. The bulk of affibody studies were performed using a variant with high affinity for the HER2/neu receptor [78].

The ABY-002 molecule labeled with 111In and 68Ga was the first affibody variant studied in clinical practice. Baum et al. found that the drugs 111In-ABY-002 and 68Ga-ABY-002 are non-toxic in BC patients and characterized by rapid elimination from normal tissues. However, whole-body scanning 1, 2, and 4 h after administration of the labeled proteins revealed their high accumulation in the liver and kidneys [61].

**Fig. 2:**
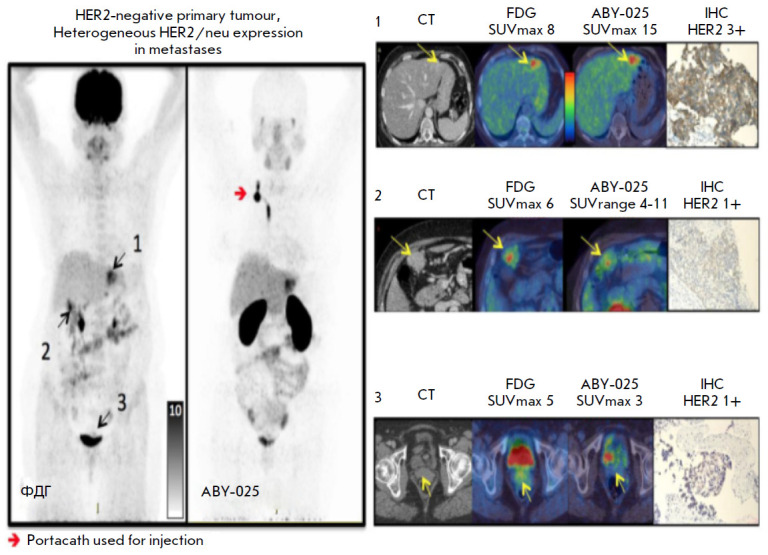
A patient with HER2-negative primary breast cancer. FDG-PET/CT detected metastases in the left lobe of the liver, peritoneal lymph nodes, and the bladder neck. The study using 68Ga-ABY-025 revealed its high additional accumulation level in hepatic metastasis and low level or no accumulation at other sites. According to IHC, the HER2/neu metastasis status is positive in the liver and negative at other sites

The second-generation modified affibody molecule ABY-025 was created by re-engineering. Sorensen et al. showed that 111In-ABY-025 is safe and can be used to differentiate the primary tumor and metastatic lesions based on the HER2/neu status in a phase I clinical trial of the compound in seven patients with locally advanced and metastatic BC (five individuals with HER2/neu overexpression; two cases with no receptor expression) [62]. However, despite promising results, limited ability to visualize small lesions in HER2- positive patients was encountered for 111In-ABY-025, which is probably due to low SPECT/CT resolution. Therefore, it was decided to study 68Ga-ABY-025 use in PET/CT. A phase I clinical study showed no toxic effects by the compound in eight patients with metastatic BC. In addition, the importance of the drug dose was confirmed, since the use of 78 μg of the protein resulted in a statistically higher drug accumulation in the liver and kidneys compared to that when using 427 μg of the protein [63]. A subsequent analysis of 16 patients with metastatic BC (12 cases with HER2/neu overexpression; four individuals with no receptor expression) showed not only the possibility of visualizing metastatic nodes (metastases to regional lymph nodes and distant organs and tissues) in all cases but also their accurate differentiation depending on the HER2/ neu status in patients with metastatic BC (*Fig. 2*) [64].

In addition, Sandberg et al. performed a study with 23 patients suffering from metastatic BC and showed that the spleen serves as the best reference organ in all modalities (followed by the blood pool and lungs) when using 111In-ABY-025 and 68Ga-ABY-025. At the same time, the tumor/spleen ratio attained a level of accuracy of 100% when separating tumor nodes, depending on the HER2/neu status 4 and 24 h after injection according to PET and SPECT, respectively [65].

The high efficiency of a labeled affibody molecule was confirmed by Xu Y. et al. In a preliminary clinical study performed in two patients, the authors showed a higher accumulation level of 68Ga-NOTA-MAL-Cys- MZHER_2:342_ in a breast tumor overexpressing HER2/ neu [79].

*ADAPTs *(*ABD-Derived Affinity Proteins*)*. *These molecules were designed using a 46-amino acid scaffold derived from the albumin-binding domain (ABD), which folds spontaneously into a three-stranded structure and is independent of disulfide bridges. Hober’s team (Royal Institute of Technology, Stockholm, Sweden) created a library that allows for synthesizing ABDs for various targets; molecules targeting various TNFα and HER3 receptors served as variants [80]. The ADAPT6 molecule, which has tropism for HER2/neu, was chosen because of its high affinity for HER2/neu (1 nM) and rapid elimination from the bloodstream thanks to low albumin binding [81].

**Fig. 3:**
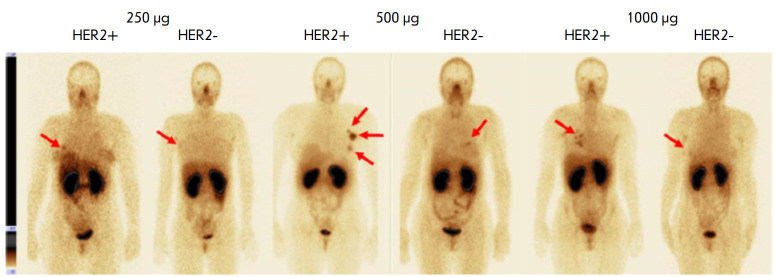
Anterior projection of planar scintigraphy of breast cancer patients with positive (HER+) and negative (HER-) expression of HER2/neu 2 h after injection of 250, 500, and 1,000 μg of 99mTc-ADAPT6 (arrows indicate a breast tumor)

Phase I clinical trials of 99mTc-ADAPT6 (9mTc; a half-life 6.01 h) included 22 BC patients with different HER2/neu expression levels in the primary tumor. Three dosages of the protein (250, 500, and 1,000 μg) were used in the study. All patients underwent wholebody planar scintigraphy and single-photon computed tomography of thoracic organs 2, 4, 6, and 24 h after administration of the labeled protein. The study results showed good tolerability of the drug and no changes in the vital organs. The most significant difference in drug distribution between the HER2/neupositive and HER2/neu-negative tumors was observed 2 h after drug injection at a dose of 500 μg (mean tumor/ background of 37 ± 19 for HER2-positive tumors and 5 ± 2 for HER2-negative tumors, *p * < 0.05, Mann– Whitney U test). The difference between the groups at other time intervals was not statistically significant. The tumor/background ratio in HER2-positive tumors was significantly higher in patients receiving the 500 µg dose than in those who received 250 and 1,000 µg (*p * < 0.05, Mann–Whitney U test). In addition, a relatively low radiation dose was set when administering 500 and 1,000 µg of the protein: 0.009 ± 0.002 and 0.010 ± 0.003 mSv/MBq, respectively, which is comparable to the data obtained in the studies of other ASPs (*Fig. 3*) [66, 82].

*DARPins *(Designed Ankyrin Repeat Proteins) are ASP members designed based on ankyrin proteins. Ankyrins are involved in the linking of membrane proteins to the cytoskeleton [83]. The DARPin backbone can include 4–6 ankyrin domains, each of which contains 33 amino acid residues; the domains are packed as two antiparallel alpha helices with a beta turn between them [84]. Considering that the molecular weight of a module is slightly over 3.5 kDa, and that DARPins consist of 4–6 modules, their molecular weight ranges from 14 to 21 kDa and is approximately a tenth the size of a conventional antibody (IgG) or a third the size of Fab [85]. Preclinical studies of DARPin variations have established their high tropism and specificity for the HER2/neu receptor [86, 87].

**Fig. 4:**
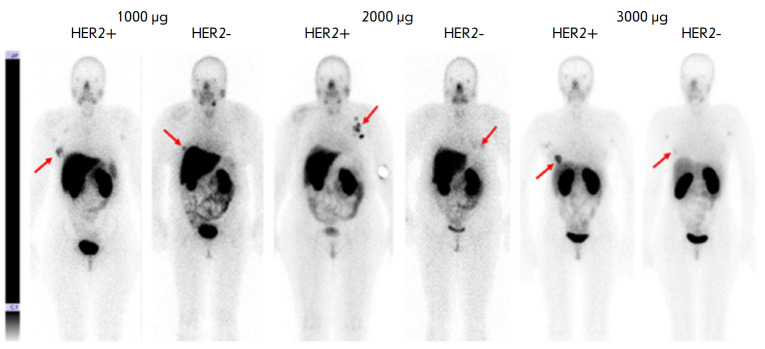
Anterior projection of planar scintigraphy of breast cancer patients with positive (HER+) and negative (HER-) expression of HER2/neu 4 h after injection of 1,000; 2,000, and 3,000 μg of 99mTc-DARPinG3 (arrows indicate a breast tumor)

Phase I clinical trials of 99mTc-DARPinG3 at a dose of 1,000, 2,000, and 3,000 µg have been performed. They included 28 BC patients with different HER2/ neu expression levels. Patients underwent wholebody planar scintigraphy and single-photon computed tomography of thoracic organs 2, 4, 6 and 24 h after drug administration. The drug ^99^mTc-DARPinG3 showed no toxic effects on the body over the entire observation period at the doses used, demonstrated rapid excretion with blood flow, and a relatively low radiation dose in patients (0.011 ± 0.001. 0.012 ± 0.006, and 0.012 ± 0.003 mSv/MBq, respectively) (*Fig. 4*). The best tumor/background ratio was observed in patients with HER2/neu overexpression in the tumor 2 and 4 h after the injection of 1,000 and 2,000 µg of the labeled protein; and 2, 4, and 6 h after the administration of 3,000 μg (*p* < 0.05; Mann–Whitney U test). At the same time, the dose of 3,000 μg turned out to be the most effective and made it possible to visualize liver metastases [67].

## CONCLUSION

The diagnosis of HER2-positive BC remains a vexing issue in clinical oncology. None of the existing diagnostic methods can fully settle the question and usually requires additional, costly, invasive, and sometimes complicated manipulations [26, 28]. This problem becomes especially evident when determining the molecular characteristics of the identified tumor nodes (metastases) and choosing the optimal level of systemic treatment.

Currently, targeted radionuclide imaging methods, which expand the possibilities of cancer diagnosis, are rapidly developing [88]. The information presented in this review allows for a more detailed look at the evolution of the radionuclide diagnostics of HER2- positive BC using various compounds as a targeting module: from full-length antibodies to a new group of small synthetic proteins, namely alternative scaffold proteins, which are present in various molecular forms with different structures, charges, and lipophilicity of the amino acid residues exposed to a solvent. The numerous preclinical studies of labeled proteins have determined the optimal characteristics of the scaffolds needed for molecular imaging, as well as their high target specificity.

To date, clinical studies of compounds based on such proteins as affibodies, ADAPTs, and DARPins for SPECT and PET have shown good tolerance, rapid elimination from the body, and the possibility to differentiate tumor lesions depending on the status of human epidermal growth factor receptor 2 HER2/ neu. An indisputable advantage of these methods over standard diagnostic approaches (FISH and IHC) is the possibility to perform simultaneous detection of additional tumor nodes and determine their molecular phenotype. The convincing results obtained in the first clinical trials point to the prospects of targeted radionuclide diagnosis and the need for further research in this direction.
